# Binding to the Minor Groove of the Double-Strand, Tau Protein Prevents DNA from Damage by Peroxidation

**DOI:** 10.1371/journal.pone.0002600

**Published:** 2008-07-02

**Authors:** Yan Wei, Mei-Hua Qu, Xing-Sheng Wang, Lan Chen, Dong-Liang Wang, Ying Liu, Qian Hua, Rong-Qiao He

**Affiliations:** 1 State Key Laboratory of Brain and Cognitive Science, Institute of Biophysics, Chinese Academy of Sciences, Beijing, China; 2 Graduate University of Chinese Academy of Sciences, Beijing, China; 3 School of Preclinical Medicine, Beijing University of Chinese Medicine, Beijing, China; Temasek Life Sciences Laboratory, Singapore

## Abstract

Tau, an important microtubule associated protein, has been found to bind to DNA, and to be localized in the nuclei of both neurons and some non-neuronal cells. Here, using electrophoretic mobility shifting assay (EMSA) in the presence of DNA with different chain-lengths, we observed that tau protein favored binding to a 13 bp or a longer polynucleotide. The results from atomic force microscopy also showed that tau protein preferred a 13 bp polynucleotide to a 12 bp or shorter polynucleotide. In a competitive assay, a minor groove binder distamycin A was able to replace the bound tau from the DNA double helix, indicating that tau protein binds to the minor groove. Tau protein was able to protect the double-strand from digestion in the presence of DNase I that was bound to the minor groove. On the other hand, a major groove binder methyl green as a negative competitor exhibited little effect on the retardation of tau-DNA complex in EMSA. This further indicates the DNA minor groove as the binding site for tau protein. EMSA with truncated tau proteins showed that both the proline-rich domain (PRD) and the microtubule-binding domain (MTBD) contributed to the interaction with DNA; that is to say, both PRD and MTBD bound to the minor groove of DNA and bent the double-strand, as observed by electron microscopy. To investigate whether tau protein is able to prevent DNA from the impairment by hydroxyl free radical, the chemiluminescence emitted by the phen-Cu/H_2_O_2_/ascorbate was measured. The emission intensity of the luminescence was markedly decreased when tau protein was present, suggesting a significant protection of DNA from the damage in the presence of hydroxyl free radical.

## Introduction

Tau is a major microtubule-binding protein that is important for the assembly and stabilization of microtubules [1]. However, tau has a higher affinity for DNA than for microtubules [2], and is found in the nuclei of human neuroblastoma cells, human cervical carcinoma cells, human macrophages, monkey kidney cells and PC12 cells [3]–[7]. The six isoforms of tau have been observed to bind to nucleolar organizer regions of acrocentric chromosomes and the fibrillar region of the nucleoli (the site for rRNA transcription) in some non-neuronal cells such as lymphocytes [5]. Greenwood and colleagues have isolated nuclei and observed tau protein covalently cross-linked to DNA, representing the binding of tau to DNA in the nucleus [8]. Recently, a functional role for nuclear tau has been proposed in relation to nucleolar organization and heterochromatinization of a portion of RNA genes [9]. As described previously, tau protein cannot bind to the nucleosome since histone displaces the bound tau from tau-DNA complex [10]. As shown by an in vitro assay (37°C), tau protein represses DNA replication, but does not affect RNA transcription in the presence of T7 RNA polymerase [11], behavior that is similar to histone. These different interactions between tau and DNA shed light on the manner in which tau associates with DNA.

In the adult human brain, there are six isoforms of tau, produced from a single gene by alternative mRNA splicing [12]–[15]. All six isoforms of tau protein contain two major domains: a projection domain (composed of an acidic region and a proline-rich domain) and a microtubule-binding domain (MTBD) [16]. The MTBD functions as a regulator of the rate of microtubule polymerization [17] and actin assembly [18], [19]. However, the role of MTBD in the association of tau with DNA was still unknown. A recent review by Holt and Koffer discusses proline-rich proteins and they emphasize the significance of the function of the proline-rich domain (PRD) [20]. But, no one has addressed the PRD of tau protein is involved in the interaction with DNA.

We have shown previously that tau protein binds to DNA forming beads-on-a-string complexes [21] and represses the disassociation of the double strand as well as improves DNA annealing [22]. So far, however, the manner of the association of tau with DNA remains a fundamental problem to be elucidated. This study is concerned with the interaction of tau protein with the double-helix to protect DNA from the impairment of hydroxyl free radical.

## Results

### Tau protein binds to a polynucleotide longer than 12 base pairs

**Figure 1:**
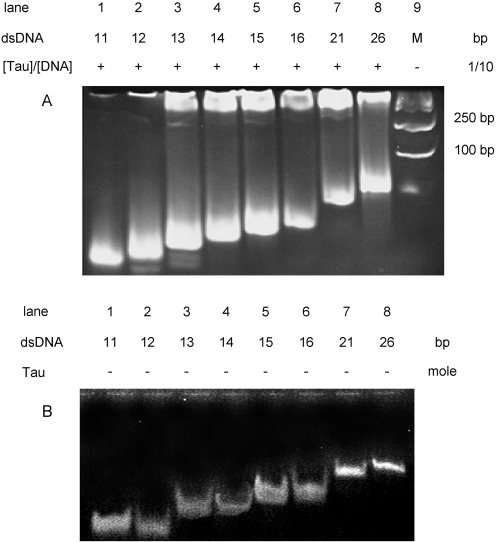
Electrophoretic mobility shift assay of double-stranded polynucleotides with different chain lengths in the presence of tau protein. Double-stranded polynucleotides (100 ng) with different chain lengths (as indicated in each lane and Table 1) were incubated with tau23 at a ratio [protein]/[DNA] of 1/10 in the buffer of 20 mM Tris-HCl (pH 7.2), containing 50 mM NaCl, 0.5 mM DTT, 1 mM MgCl_2_ and 0.5 mM EDTA at room temperature for 30 min, and then aliquots were taken for electrophoresis on a nondenaturing 20% polyacrylamide gel (A). The polynucleotides without tau protein were used as controls under the same experimental conditions (B).

**Figure 2:**
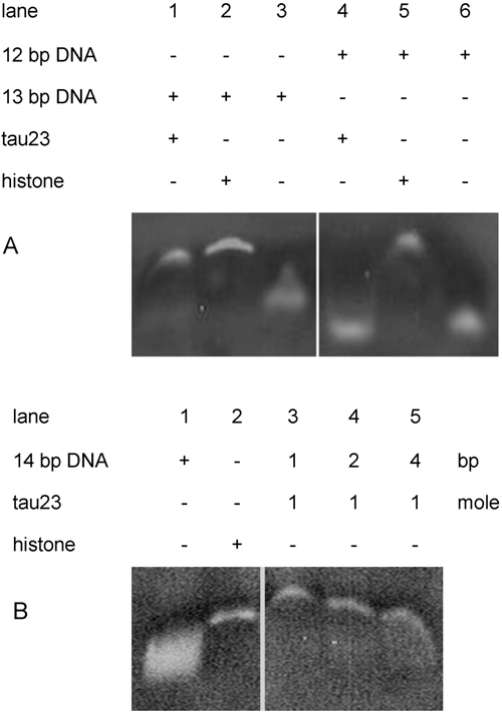
Analysis of the complexes of tau protein and DNA by EMSA. Polynucleotides (12 and 13 bp) were incubated with tau protein under the same experimental conditions (A) as described in Figure 1. 14 bp DNA and tau at different quantitative ratios are as indicated (B).

The electrophoretic mobility shift assay (EMSA) is commonly used to investigate the interaction of proteins with polynucleotides. To investigate the minimum polynucleotide that is bound to tau protein, we synthesized a series of polynucleotides (11–26 bp) and incubated them with tau protein for EMSA (Table 1). The mobility of DNA bands was reduced in the presence of tau protein (Figure 1A). This effect was particularly marked for polynucleotides of 13 bp or greater in length. Under these experimental conditions, no reduction in mobility of the polynucleotides was observed in the absence of tau (Figure 1B). To investigate further whether the protein discriminated between 13 bp and other lengths of polynucleotides, 12 bp and 13 bp polynucleotides were incubated with tau and subjected to EMSA. A band shift for the 13 bp DNA incubated with tau was observed, but not for 12 bp DNA under the same conditions (Figure 2A). As shown in Figure 2B, the incubation of 14 bp DNA with tau protein at different concentrations showed that the degree of retardation depended upon the quantitative ratio between the protein and DNA (the ratio is defined in Materials and Methods). This suggests that tau protein prefers binding to a 13 bp or a longer polynucleotide.

**Table 1:** Double stranded polynucleotides synthesized.

Number of base pairs	Nucleotide sequence
26 bp	5′actccgtgcataaataataggcactcg3′
	3′gaggcacgtatttattatccgtgagcc5′
21 bp	5′gctccgtgcataaataataggc3′
	3′gaggcacgtatttattatccgagc5′
16 bp	5′actccgtgcataaataa3′
	3′gaggcacgtatttatt5′
15 bp	5′atccgtgcataaataa3′
	3′aggcacgtatttattc5′
14 bp	5′accgtgcataaataa3′
	3′ggcacgtatttattc5′
13 bp	5′acgtgcataaataa3′
	3′gcacgtatttattc5′
12 bp	5′agtgcataaataa3′
	3′cacgtatttattc5′
11 bp	5′actccgtgcata3′
	3′gaggcacgtatc5′

**Figure 3:**
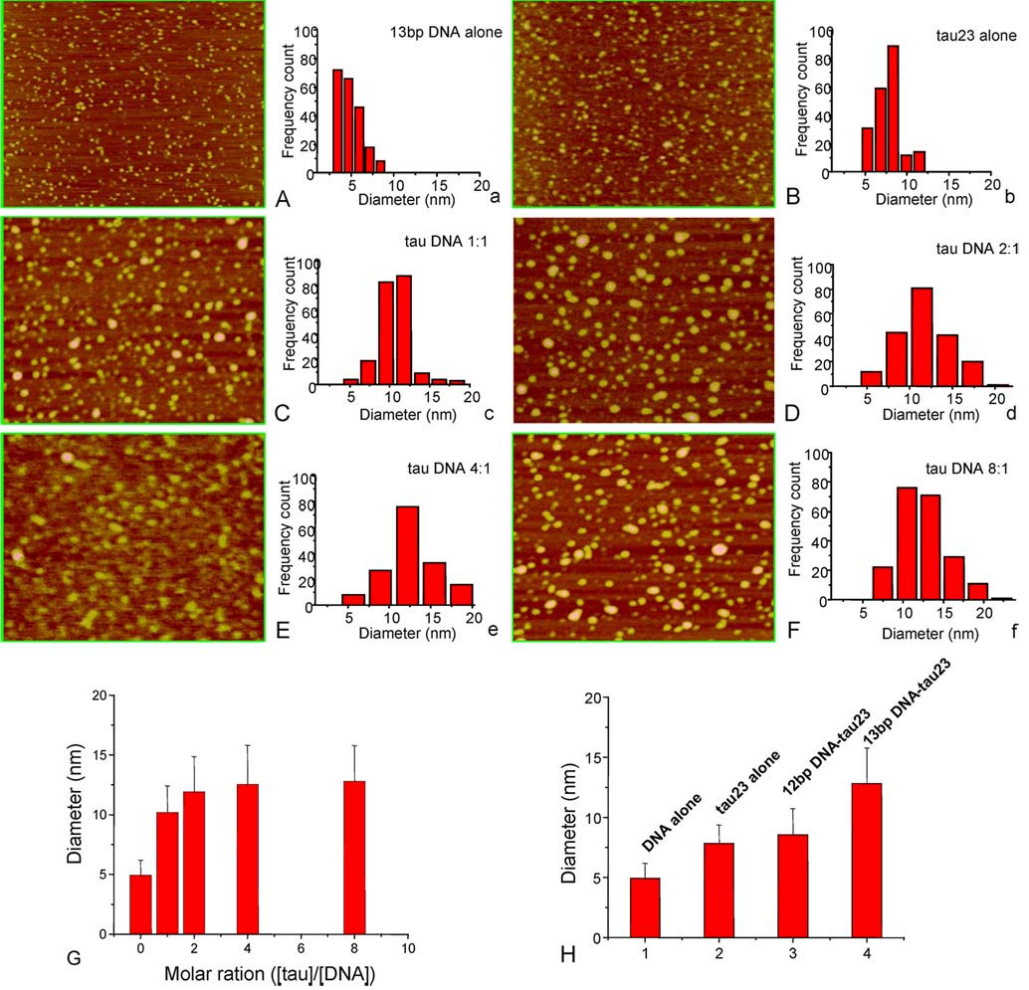
Tau protein, 12 and 13 bp DNA and their complexes under AFM. A 13 bp DNA (A), tau protein (B) and their complexes at different ratios as indicated (C–F) were imaged by tapping-mode AFM. The histograms (a–f) are frequency counts of the AFM data in panels A–F and show the population distribution of sizes of the particles. Panel G shows the change in average size (horizontal diameter) of 13 bp DNA-tau23 complexes at different quantitative ratios. The average size (nm, ordinate) of the particles of DNA in the presence or absence of tau ([tau]/[DNA] = 8/1) is shown (H).

In order to confirm the results of EMSA, atomic force microscopy (AFM) was used to observe the interaction of tau with 12 bp and 13 bp DNA. We measured the sizes of the particles of samples at the ratios [protein]/[DNA] of 1/1, 2/1, 4/1 and 8/1 (0.5 µM DNA, Table 2). 13 bp DNA alone (0.5 µM) and tau alone (10 µM) were used as controls. At the quantitative ratio of 1/1 (Figure 3C), the particle size of 13 bp DNA-tau complex was 10.19±2.22 nm, which was larger than for 13 bp DNA (4.93±1.25 nm, Figure 3A) or tau alone (7.82±1.55 nm, Figure 3B). The sizes of the 13 bp DNA-tau complexes were different for each of the [protein]/[DNA] ratios 2/1, 4/1 and 8/1 (Figure 3D–F). The horizontal diameter increased with increasing quantitative ratio. However, the size did not further increase when the ratio became higher than 4 (Figure 3G), suggesting saturation of binding. Under the same conditions, the particle size of 12 bp polynucleotide incubated with tau (8.54±2.19 nm) was not significantly increased, compared to the size of tau23 alone (Figure 3H). This supports the EMSA result that a 13 bp polynucleotide is long enough for tau to bind.

**Table 2:** Sizes of complexes of tau23 bound to DNA measured by AFM.

Particles	Mean (nm)	SD	Number
Tau-12 bp DNA 8∶1	8.54	2.19	205
Tau-13 bp DNA 1∶1	10.19	2.22	210
Tau-13 bp DNA 2∶1	11.91	2.96	200
Tau-13 bp DNA 4∶1	12.54	3.27	160
Tau-13 bp DNA 8∶1	12.81	2.96	210
13 bp DNA alone	4.93	1.25	210
12 bp DNA alone	4.81	1.32	212
Tau23 alone	7.82	1.55	205

The software provided with the NanoScope (R) III instrument was used to measure the horizontal diameters of the particles.

### Tau protein binds to the minor groove of DNA double-strand

**Figure 4:**
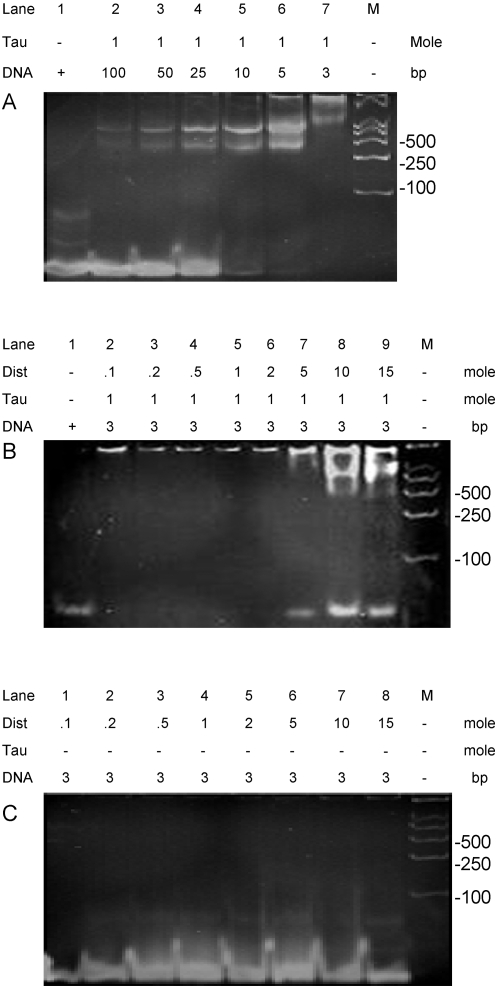
A minor-groove binder competing with tau protein in binding to DNA. Conditions were as described in Figure 1, except 26 bp dsDNA was used with the addition of distamycin A, a minor groove binder (A). The DNA was mixed with tau protein at different quantitative ratios as indicated. Distamycin A at different concentrations (quantitative ratios as indicated) was added to the tau-DNA complex (B). DNA in the presence of distamycin A at different concentrations was used as a control (C). M represents molecular mass marker.

Double-stranded DNA (dsDNA) contains major and minor grooves. Consequently, distamycin A (a minor groove binder) was employed as a competitor to assay whether tau protein bound to the minor groove of DNA [23]. As shown in Figure 4A, the migration of 26 bp DNA bands became retarded in the presence of tau when the [tau]/[DNA] ratio was 1/100. The band shift of the 26 bp DNA was dependent upon the quantitative ratio [tau]/[DNA]. At the quantitative ratio of 1/3 leading to the greater band shift (lane 7, Figure 4A), different concentrations of distamycin A were added to the tau-DNA complexes at room temperature (RT) for 30 min to compete with the bound tau. When the concentration of the minor groove binder was increased to the quantitative ratio [distamycin A]/[DNA] of 5/3, a non-retarded DNA band appeared at the frontier of the gel, suggesting that some bound DNA was released from the tau-DNA complex (lane 7, Figure 4B). More DNA was released when the quantitative ratio [distamycin A]/[DNA] was increased to 10/3 or 5/1. Distamycin A alone at different concentrations as control did not cause any marked DNA retardation in the gel (Figure 4C), showing that the association of distamycin A itself did not cause a change in retardation of the DNA bands. This suggests that tau binds to the minor groove of the double strand.

**Figure 5 pone-0002600-g005:**
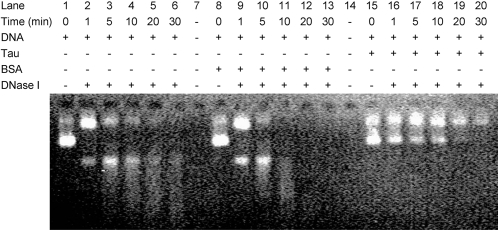
DNA hydrolysis with DNase I in the presence of tau protein. Tau protein and pEGFP-N1 DNA (quantitative ratio, 1/8) were incubated in 20 mM Tris-HCl buffer containing 2 mM MgCl_2_ (pH 8.3, RT, 30 min), and then DNase I (0.05 units) was used to hydrolyze DNA (4730 bp, 100 ng) at 37 °C. Aliquots were taken for agarose gel electrophoresis at different time intervals as indicated. Five mM EDTA (final concentration) was employed to stop the enzymic reaction (lanes 15–20). Hydrolyses of DNA alone (lanes 1–6) and DNA in the presence of BSA (lanes 8–13) were carried out as controls.

DNase I, which is known to bind to the minor groove of B-type DNA [24] was also employed to assay the binding of tau protein to dsDNA. The binding of tau should compete with DNase I during DNA hydrolysis if the protein binds to the minor groove. As shown in Figure 5 (lanes 15–20), in the presence of tau protein, the DNA was not significantly digested when it was incubated with DNase I for 10 min. However, the DNA was almost totally hydrolyzed in 10 min in the absence of tau protein (lanes 1–6). Under the same experimental conditions, hydrolysis of DNA in the presence of BSA occurred rapidly and the DNA band disappeared within 5 min (lanes 8–13). This indicates that tau protein binds competitively to the minor groove and interferes with the hydrolysis of DNase I.

**Figure 6:**
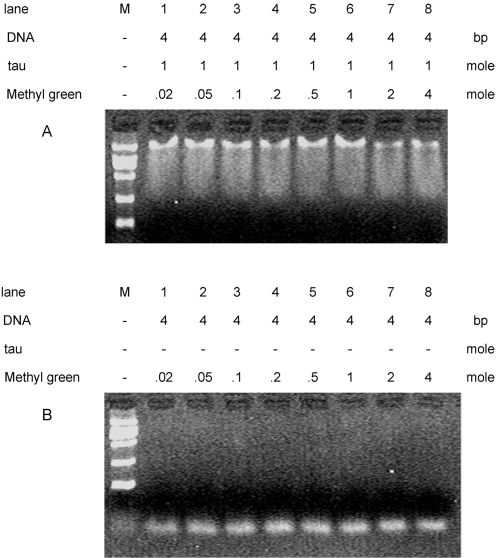
Electrophoretic mobility shift assay of tau23-DNA complexes in the presence of methyl green. Conditions were referred to Figure 4, except methyl green a major groove binder was used instead of distamycin A. Methyl green at different concentrations was added to the 26 bp DNA-tau23 complex (A). 26 bp DNA in the presence of methyl green at different concentrations was used as a control (B). M represents molecular mass marker.

Now that tau protein has shown to insert the minor groove of the DNA, we are concerned whether this protein could also bind to the major groove of the double-strands. Thus, methyl green, a DNA major groove binder [25], [26] was used as a major groove binding competitor for this competitive experiment. As shown in Figure 6A, in the presence of methyl green, no distinct effect on DNA band shifting could be observed on the agarose gel. The results depicted that this major groove binder did not affect the shifting of tau-DNA complex. That is to say, although methyl green blocked the major groove, tau protein is still able to associate with the DNA. This suggests again that tau protein inserts the minor groove of the double strands.

### The microtubule-binding domain and the proline-rich domain of tau are involving in DNA association

We next investigated which domain of tau is involved in DNA binding. For this, four truncated mutants were constructed with different regions and domains from tau23: the N-terminal region (τN), the proline-rich domain (τPro), the microtubule-binding domain (τMTBD) and the C-terminal region (τC). The four truncated mutants were expressed in *E. coli*, under the induction of IPTG. Each of the proteins showed a single band in SDS-PAGE after purification (not shown).

**Figure 7:**
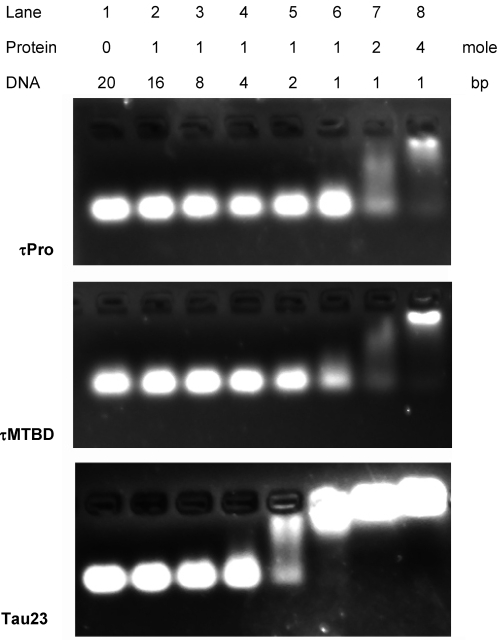
20 bp DNA in the presence of tau23 and its mutants on PAGE. Twenty bp DNA (final concentration 2.5 µM) was incubated with tau23, τPro or τMTBD at different quantitative ratios as indicated in the panels (RT, 30 min). Aliquots were taken for analysis by 20% PAGE. The double-stranded polynucleotide is: 5′AACGAGAAGCGCGATCACAT3′/3′TTGCTCTTCGCGCTAGTGTA5′.

In EMSA (Figure 7A), a distinct band shift of DNA was observed in the presence of τPro at [protein]/[DNA] ratios of 2/1 or higher. Similarly, DNA also showed band retardation when it was incubated with the τMTBD (Figure 7B). Under the same conditions, neither of the mutants τN or τC showed any effect on the DNA band mobility in the gel (not shown). This suggests that the PRD and MTBD are individually involved in DNA binding. In the presence of intact tau23, DNA started to show a marked band shift at a ratio of [protein]/[DNA] 1/2 under these experimental conditions (Figure 7C), four times lower than the ratio for τPro or τMTBD to induce a marked band shift of DNA. The lower quantitative ratio (1/2) indicates that intact tau has a stronger affinity for DNA than τPro or τMTBD. It appears that both PRD and MTBD of the intact tau protein are involved in the interaction with dsDNA.

**Figure 8:**
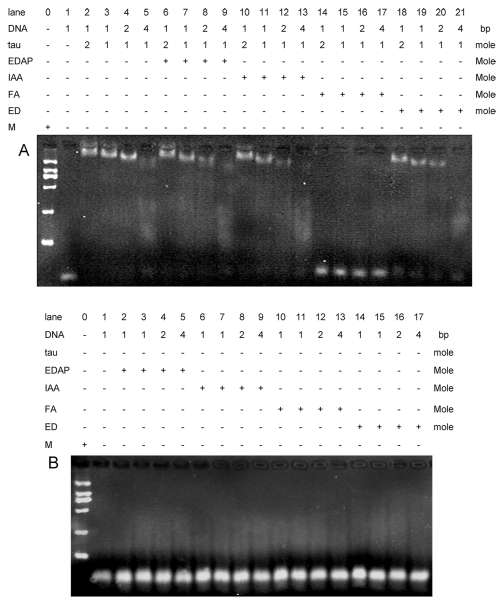
The binding of DNA to tau23 protein that was modified with EDAP, IAA, formaldehyde and ethanedial, respectively. The aminoacid residues of Lys were modified with formaldehyde (FA), Cys with iodoacetic acid (IAA), Arg with ethanedial (ED), Asp and Glu with 1-ethyl-3-(3-dimethylaminopropyl (EDAP), respectively, as described under Materials and Methods. After that, the modified tau protein was incubated with 26 bp DNA as mentioned in Figure 1, followed by aliquots were taken for EMSA. The ratios of [protein]/[DNA] are as indicated.

To clarify what amino acid residues of tau protein are involved in the association with DNA, we have blocked the ε-amino group (Lys) with formaldehyde, the thial group (Cys) with IAA, the carboxyl groups (Asp and Glu) with EDAP, and the guanidine groups (Arg) with ethanedial (ED), respectively [27]. Then, we incubated the modified tau protein with DNA and then took the aliquots for electrophoresis. As shown in Figure 8A, only formaldehyde modified tau becomes unable to bind to DNA, but the modification of tau protein with IAA, EDAP, and ED could not result in a marked effect on the mobility of the DNA bands. The DNA suspended with the modifiers FA, ED, IAA and EDAP in the absence of tau protein used as controls had no effects on the DNA bands mobility. Notably, similar EMSA results were obtained when the modified τPro and τMTBD were incubated with DNA (data not shown). The mutants τN and τC had no effect on the DNA band mobility. As we know, formaldehyde blocks ε-amino groups of Lys and thus disturbs the interaction of tau protein with DNA. It demonstrates that Lys residues are essentially involved in the association of tau protein with DNA.

**Figure 9:**
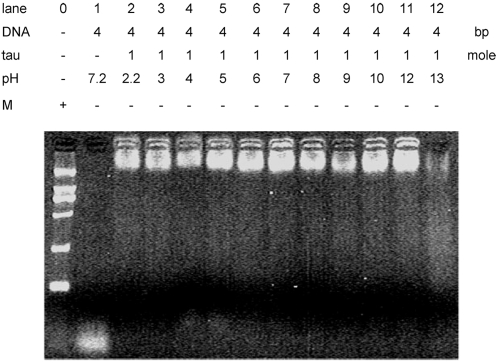
Electrophoretic mobility shift assay of tau23-DNA complexes at different pH values. Conditions were as for Figure 1, except 26 bp DNA was incubated with tau23 at different pH values.

The interaction of a Lys residue with a nucleotide is mostly dependent upon electrostatics and to form hydrogen bond between ε-amino group and DNA bases [28]. To study the interaction of tau with DNA, we carried out EMSA of tau with DNA at different pH values from 2.2 to 13. As shown in Figure 9, the retardation of tau-DNA complex is markedly disturbed at the pH higher than 12. In fact, the pK of ε-amino group of Lys is 10.8 and its positive charge is counteracted when the pH value increases. This experiment further demonstrates that Lys is at least the essential residue for tau protein to associate with DNA.

### The binding of tau protein protects DNA from the damage of peroxidation

**Figure 10:**
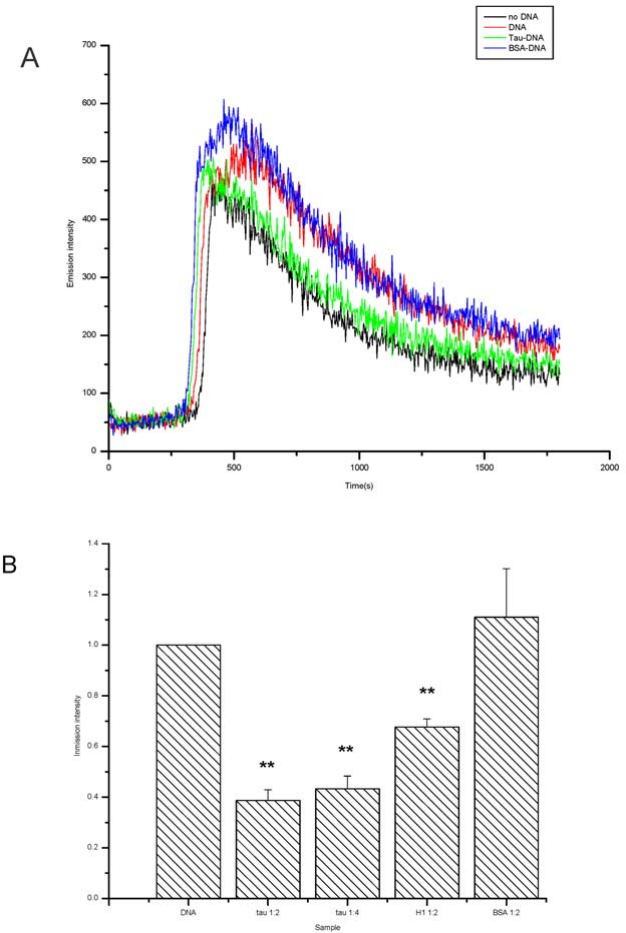
Prevention of DNA from peroxidation. Copper, ascorbate and 1,10-phenanthroline were premixed in 0.1 M NaOAc/HOAc (pH 5.2) buffer; tau at different concentrations was premixed with DNA at 37 °C for 30 min. Samples were incubated with phen-Cu/ascorbate at room temperature for 5 min. Afterwards, H_2_O_2_ was added to the solution to give a final volume of 1.2 ml. The chemiluminescence produced in the phen-Cu/H_2_O_2_/ascorbate system was immediately recorded with a computerized high-sensitivity single-photon counter. The voltage in the photomultiplier was kept at 1000 V (** P<0.01).

What the effect of tau on DNA is so far brought up since this protein is experimentally bound to the minor groove of DNA double strands. Hydroxyl free radical is one of the most important factors resulting in DNA damage [29]. Thus, we have investigated whether tau protein is capable of preventing DNA from the impairment by hydroxyl free radical in vitro. We employed the phen-copper complex as a reporting model, described previously by Ma et al. [30]. Hydroxyl free radical (⋅OH) impairs DNA, resulting in DNA chain broken, which leads to an increase in the emission intensity of the chemiluminescence produced from the phen-Cu/H_2_O_2_/ascorbate system. As depicted in Figure 10, the luminescent intensity was markedly decreased when tau protein was added. The decrease in the luminescent intensity is dependent upon the concentration of tau in the reaction system. Under the same conditions, histone I as a positive control shows the same protecting effect on DNA, but as a negative control BSA does not. That is to say, the damage of DNA is notably decreased in the presence of tau protein under the attack of hydroxyl free radical.

## Discussion

Tau protein was first found to associate with tubulin and promote microtubule assembly [31], [32]. To date, a great deal of work on the interaction of tau with microtubules has been reported [33]–[35]. However, some observations indicate that tau protein localizes on acrocentric chromosomes in the nucleolar regions and binds to DNA in nucleus [5], [7], suggesting that tau is a multifunctional protein. To study what role of tau protein plays in the interaction with DNA, we have previously observed the effect of tau on DNA conformations and our results show that the protein is capable of preventing thermal denaturation of dsDNA and improving DNA annealing [11], [22]. That is to say, tau protein stabilizes DNA double strands. In this work, as mentioned above, we have found that tau protein is a DNA minor groove binder that protects DNA under the attack of hydroxyl free radical.

First, to investigate the manner of tau binding to DNA, we should determine what the basic chain-length of a DNA can be associated with tau protein. Thus, we synthesized DNA in different chain lengths from 9 to 26 base pairs. We have observed that tau protein is bound to a 13 bp polynucleotide, whereas shows no or at least much weaker potential to associate with a 12 bp or shorter dsDNA. Using EMSA, a band shift for 13 bp polynucleotide was markedly observed, but not for 12 bp DNA. As shown in Figure 2B, the association of 14 bp DNA with tau protein also causes retardation of the DNA band mobility in the gel. Furthermore, as observed by AFM, the size of the complex of 13 bp DNA-tau is clearly larger than that of the mixture containing 12 bp DNA and tau, indicating 12 bp DNA and tau are separately on the mica surface. This indicates that tau and 13 bp DNA are associated to form complexes. The results from EMSA also show a marked retardation of 13 bp DNA band in the presence of tau on the gel, but not 12 bp DNA; that is, a 13 bp DNA is a basic motif for tau protein to bind.

Second, tau protein is demonstrated binding to the minor groove of dsDNA without nucleotide sequence specificity (Figures 4–6). This view is supported by the following observations: (1) Displacement by distamycin A and competition with DNase I indicate that the minor groove of the DNA double helix is the binding site for tau protein. (2) As mentioned above, the major groove binder methyl green does not markedly compete with tau protein in the association with DNA. (3) Some important nucleic associated proteins, such as histone H2b and H3 recognize the minor groove binding to DNA without sequence specificity [36], [37]. (4) Our previous studies have shown that tau protein binds non-specifically to different sequences of both eukaryotic (bovine thymus) and prokaryotic (plasmid) DNA [22]. (5) According to a recent study, if a protein binds to the major groove, it should associate with DNA in a sequence specific manner [38]–[40].

**Figure 11:**
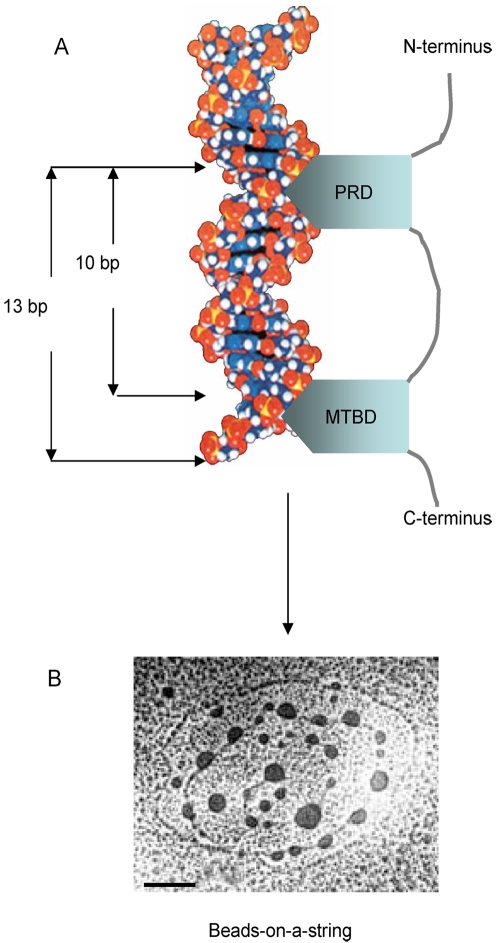
A hypothetic model of the tau-DNA interaction. The proline-rich domain and the microtubule-binding domain may bind to the DNA minor groove in the same orientation plane (A). The complex of the tau protein with double-stranded DNA has a beads-on-a-string appearance under the electronic microscope (B). Bar = 100 nm.

For B-type DNA, 10 base pairs make an entire DNA double helix. Tau protein, however, preferably bound to a 13 bp or longer polynucleotides, suggesting a special mode of binding between tau and DNA. As mentioned above, the affinity of the mutant τPro or τMTBD was much lower than the intact tau protein. It appears that tau could be bound to a 12 bp or shorter DNA unless the protein concentration is high enough. This implies a simultaneous association of both PRD and MTBD with the dsDNA. If the two domains bound to the minor groove in different orientations, as found for a POU box protein whose homeodomain and POU-specific domain bind to a DNA octamer [41], tau protein should strongly associate with a shorter length of dsDNA (<10 bp). As illustrated in Figure 11A, we hypothesize here that both PRD and MTBD may associate simultaneously with the minor groove of dsDNA within the same plane. The data which support this proposed model are as the following: (1) Tau protein binds to a 13 bp or longer DNA. In the case of a 10 bp polynucleotide, its minor groove may not be long enough for PRD and MTBD to bind at the same time in the same orientation plane. That is to say, when the proline-rich domain occupies the minor groove, there is not sufficient minor groove within a 10 bp polynucleotide to accommodate the microtubule-binding domain in the same orientation and vice versa. Nonetheless, a 13 bp (or a longer) polynucleotide is long enough to provide binding sites for the two domains. (2) The association of tau bends DNA double-strand as shown in Figure 11B. If the two domains bound to DNA in different orientations, the DNA strands would not be bent.

Tau23 is composed of 1 Cys, 18 Glu, 14 Arg, 10 His, and 36 Lys residues. As mentioned above, modification of Cys, Glu, Arg and His did not affect the interaction of tau with DNA. This indicates that those amino acid residues are not involved in the binding of tau to DNA. In the presence of formaldehyde, however, the association of tau protein with DNA is suppressed. Formaldehyde reacts with α-/ε-amino groups of a protein [42]–[44]. That is to say, Lys is the essential residue for tau to interact with DNA. Furthermore, we have constructed the mutants τN and τC and suspended them with DNA. We could not observe the association of the two mutants with DNA measured on EMSA. This suggests that the Lys residues out of the PRD and MTBD are not related to the DNA binding. The Lys residues of PRD and MTBD are essential to interact with the minor groove of DNA. Although the PRD (7 Lys) and MTBD (12 Lys) contain 19 Lys residues, some of them are supposed to be involved in the interaction between tau and DNA. This viewpoint is based on the following points: (1) the pK of the ε-amino group of Lys is 10.8 interacting with DNA bases in electrostatics; (2) the interaction between tau and DNA is markedly suppressed at the pH higher than 12, showing dependent upon the electrostatics measured by EMSA; and (3) modification with formaldehyde interferes with the association of tau with DNA.

DNA damage is an extremely important event during cell dysfunction or apoptosis [29]. Hydroxyl free radical attacks and impairs DNA, lipid, and protein. We have employed 1,10-phenanthroline and copper in the presence of ascorbate to investigate the DNA damage and the protect ability of tau. The reaction of 1,10-phenanthroline with hydrogen peroxide in the presence of copper results in chemiluminescence. The ⋅OH radical produced from the reaction impairs and breaks DNA, leading to an increase of the chemiluminescence. As mentioned above, prevention of DNA from the impairment by ⋅OH depends on tau concentration (Figure 10B). Similar result could be seen when histone I was used as a positive control. Protection of histone on DNA has been reported by Richter C and Ozawa T [45], [46]. This suggests that tau protein protects DNA through its blocking the attack of hydroxyl free radical. However, whether tau protein is able to protect DNA in vivo needs further investigating.

Wojtuszewski K and Mukerji I have studied the interaction of HU with DNA using UV resonance Raman spectroscopy [47]. They observed that *Anabena* HU binds to the minor groove and bends the DNA double-strand. Another DNA chaperone HMGB1 also bends the double-strand and binds in a beads-on-a-string manner without sequence specificity [48]. Tau protein seems to act in a DNA chaperone-like manner in the association with the DNA double-strand. Data that support this viewpoint are as follows: (1) Like DNA chaperones such as HU and HMGB1, tau protein binds to the minor groove of DNA, without nucleotide sequence specificity. (2) The association of tau with DNA bends the double-strand in a beads-on-a-string manner as shown in Figure 11B. (3) Tau protein prevents the DNA double helix from disassociating during thermal denaturation, and improves its renaturation, indicating the marked effect on refolding of the DNA double-strand [22]. These observations suggest that the interaction of tau protein with DNA is similar to a DNA chaperone. However, the interaction between tau and DNA in a high resolution (crystal structure) remains to be further investigated.

## Materials and Methods

### Truncated tau mutant constructions

Prokaryotic expression vector (Prk172) bearing the gene of human tau23 was a gift from Dr. Goedert [12]–[14]. As described before [49], we designed a series of primers to construct four truncated tau mutants with different domains of tau23: τN (aa1-113, the N-terminal region), τPro (aa114-193, the proline-rich domain), τMTBD (aa198-278, the microtubule-binding domain) and τC (279–352, the C-terminal region). The fragments of the tau DNA were prepared by PCR or megaprimer PCR amplification and subcloned into the prokaryotic expression vector pET-28a(+) (Novagen, Germany) with a His-tag on the C-terminus. The clones of these constructs were transformed into *E. coli* BL21 (DE3) cells for protein expression. Neuronal tau23 and the truncated mutant proteins (τN, τPro, τMTBD and τC) were expressed and purified as described previously [10], [49]. Sephadex G50, Q-Sepharose and SP-Sepharose columns were from Pharmacia (Pfizer, Canada). The purified tau23 and the truncated mutant proteins yielded a single protein band on SDS-PAGE (not shown). The bicinchoninic acid (BCA) protein-assay kit [50] to determine the protein concentration was the product of Pierce Biotechnology Inc (USA). The other reagents were analytic grade and used without further purification.

### Electrophoretic mobility-shift assay (EMSA)

A series of polynucleotides (from 11 bp to 26 bp) were synthesized (Shanghai Sangon Co., China) according to the nucleotide sequence of mouse N-Oct-3 (Table 1) [51]. For effective annealing, one more nucleotide was added at each 5′ terminus [10]. The polynucleotides were denatured by heating to 95 °C in a water bath for 5 min, and then complementary strands were annealed by cooling slowly down to room temperature. The concentration of the annealed dsDNA (11, 12, 13, 14, 15, 16, 21 and 26 bp) was determined according to the absorbance at 260 nm on a Hitachi U-2010 UV Spectrophotometer (Japan).

Tau protein was incubated with 100 ng dsDNA at the ratio [tau]/[DNA] = 1/10 in 10 µl TNME solution with 20 mM Tris buffer (pH 7.2), 50 mM NaCl, 0.5 mM DTT, 1 mM MgCl_2_, and 0.5 mM EDTA (RT, 20 min). The quantitative ratio of [tau]/[DNA] was defined as the ratio of the number of protein molecules to the number of DNA base pairs, unless otherwise stated. The dsDNA-tau complexes were resolved by non-denaturing 20% polyacrylamide gel and electrophoresed at 100 V in 1×TBE running buffer (89 mM Tris base, 89 mM boric acid, and 2 mM EDTA, pH 8.3) at 4 °C for 2 h. After that, the gel was stained with EB (0.5 µg/ml) for 30 min and visualized using a gel UVP Image store 7500 system (Dingyong Co., China).

### Minor-groove competition assay

To determine an optimal quantitative ratio of [tau]/[DNA] at which the DNA showed a marked retardation in the gel, 26 bp dsDNA (100 ng/10 µl) was incubated with tau in TNME solution. The quantitative ratios of [tau]/[DNA] were: 0, 1/100, 1/50, 1/25, 1/10, 1/5, and 1/3. After the incubation, the mixtures were electrophoresed on an 8% non-denaturing polyacrylamide gel in 1×TBE buffer. Nucleic acids in the gel were stained with SYBR-Green I (a nucleic acid gel stain, Molecular Probe Co., USA) for 30 min, and then observed under an ultraviolet scanner (Amersham Pharmacia Biotech, Sweden). A minor groove binder distamycin A (Sigma, USA) at different concentrations was used as a competitor. Tau protein was incubated with DNA at the optimal quantitative ratio (1/3) at room temperature for 30 min, and then different concentrations of distamycin A were added. Aliquots (10 µl) were taken for electrophoresis. DNA incubated with distamycin A in the absence of tau was used as control.

On the other hand, methyl green, a DNA major groove binder at different concentrations was used as a negative control. The reaction was performed in the same TNME buffer and then aliquots were taken for EMSA.

### Hydrolysis of DNA with DNase I in the presence of tau protein

Tau protein and pEGFP-N1 DNA (quantitative ratio, 1/8) were incubated in 20 mM Tris-HCl buffer, containing 2 mM MgCl_2_, (pH 8.3, RT, 30 min), and then DNase I (0.05 units, Sigma, USA) was used to hydrolyze DNA (4730 bp, 100 ng) at 37 °C. Aliquots were taken for agarose gel electrophoresis at different time intervals as indicated (Figure 5). EDTA (final concentration 5 mM) was employed to stop the enzymic reaction. Hydrolysis of DNA alone or DNA in the presence of BSA was carried out as control.

### Modification of amino acid side groups of tau23 with different modifiers

To investigate what amino acid residues of tau protein are involved in the association with DNA, chemical modifiers were employed in the experiments as described previously [27]. Iodoacetic acid (IAA, Sigma, USA) resuspended in 0.2 M acetic acid-sodium buffer (pH 5.6) was used for blocking thial group; 1-ethyl-3-(3-dimethylaminopropyl (EDAP, sigma, USA) in 0.2 M acetic acid-sodium buffer (pH 5.6) was for carboxyl group; formaldehyde (Sigma, USA) in 25 mM phosphate buffer (pH 7.2) was for ε-amino groups; and ethanedial (Sigma, USA) in 25 mM phosphate buffer (pH 7.2) was for the guanidinium group of Arg. Modifiers were added to tau23 at the ratio ([reagent]/[protein]) of 100∶1 at 37°C for 24 hours. The modified tau protein was added to 26 bp DNA for EMSA as mentioned in Figure 1. Modifiers in the absence of protein were added to 26 bp DNA as controls.

### Analysis by atomic force microscopy

All of the solutions used were filtered through a 0.22 µm filter. Samples were diluted to the desired concentration using phosphate buffer 25 mM Na_2_HPO_4_-NaH_2_PO_4_ (pH 7.2), containing 2 mM MgCl_2_ and 5 mM NaCl. The solution of magnesium chloride (2 mM) was used to enhance the adsorption of DNA onto the mica surface. The samples (10 µl) were kept at room temperature for 10 min to allow adsorption onto the mica. Observation under atomic force microscopy (Mutiplemode-I, Digital Instruments, USA) was as described previously [42].

### Analysis by electron microscopy

15 µl of pET-15b (15 µg, in 10 mM Tris-HCl and 1 mM EDTA, pH 7.2) was added to 25 µl of tau (35 µg, in 25 mM Na_2_HPO_4_ - NaH_2_PO_4_, pH 7.2). The mixtures were incubated (RT, 30 min) and then fixed with 0.3% glutaraldehyde (RT, 10 min, Sigma, USA). The samples were separated from free proteins by filtration through a 2-ml column of Sepharose CL-6B (Pharmacia Biotech, UK) equilibrated with 10 mM Tris-HCl, pH 7.9. The filtrate was adsorbed onto glow-discharged thin carbon coated 300 mesh copper grids, dehydrated through a graded water-ethanol series, fixed with 0.2% glutaraldehyde (RT, 20 min), and rotary shadow-cast with Pt. Samples were visualized under a JEOL JEM-100CX electron microscope (JEOL Ltd., Japan).

### Determination of the effect of tau on the antioxidation

According to Ma et al. [30], copper, ascorbate and 1,10-phenanthroline were premixed in 0.1 M NaOAc/HOAc buffer (pH 5.2); different concentrations of tau were premixed with DNA at 37 °C for 30 min. Samples were incubated with phen-Cu/ascorbate at room temperature for 5 min. Afterwards, H_2_O_2_ was added to the solution to give a final volume of 1.2 ml. The chemiluminescence produced in the phen-Cu/H_2_O_2_/ascorbate system was immediately recorded with a computerized high-sensitivity single-photon counter (type BPCL-4, manufactured in the Institute of Biophysics, Chinese Academy of Sciences, China). The voltage in the photomultiplier was kept at 1000 V.
